# Organizational Factors to Reattract Nurses to Hospital Employment

**DOI:** 10.1001/jamanetworkopen.2025.56570

**Published:** 2026-02-09

**Authors:** Karen B. Lasater, Matthew D. McHugh, K. Jane Muir

**Affiliations:** 1 Center for Health Outcomes and Policy Research, School of Nursing, University of Pennsylvania, Philadelphia; 2 Leonard Davis Institute of Health Economics, University of Pennsylvania, Philadelphia

## Abstract

This cross-sectional study examines survey responses from registered nurses who left their job in the past 5 years regarding organizational factors likely to reattract them to hospital employment.

## Introduction

US hospitals have a retention and recruitment crisis of registered nurses (RNs).^1^ Consensus among major nursing organizations is that the retention crisis is a problem that cannot be resolved by training more RNs.^2^ Continuing to expand the nursing workforce without addressing the underlying reasons why nurses leave hospital employment (and what would reattract them) is akin to fueling a leaking gas tank.^3^ In this study, we examine what factors, if addressed by employers, would be likely to reattract RNs to hospital employment.

## Methods

This cross-sectional study used data from Nurses4All survey of RNs in 10 states. RNs were included if they reported (1) their employment status as employed but not in health care, not currently employed, or retired; (2) their most recent job was as a hospital staff RN; and (3) they had left their job in the past 5 years (2023 to 2019). Data on RNs’ age, employment, and career experiences (eg, satisfaction with career, years worked as RN, and likelihood of returning to work as RN) were collected. RNs responded to the question, “What would increase your likelihood of return to work as a nurse?” by selecting all that apply from a list. The study was approved by the UPenn institutional review board and conforms to the STROBE reporting guidelines. Informed consent was acknowledged through respondents’ participation in the study. Additional methods are available in the eAppendix in Supplement 1.

## Results

Of 4043 RNs who left a hospital job in the past 5 years and were not currently working in health care, 340 (8%) reported being employed outside health care, 1438 (36%) were not currently employed, and 2265 (56%) were retired (Table 1). A total of 3197 RNs (79%) reported being very or moderately satisfied with nursing as a career, with high satisfaction among 2032 retired RNs (90%).

**Table 1.** Demographics and Employment Experiences of Registered Nurses Not Employed in Health Care^a^

Measure	No. (%) of nurses^b^			
	Total (N = 4043)	Employed but not in health care (n = 340)	Not currently employed (n = 1438)	Retired (n = 2265)
Demographics				
Age, mean (SD), y	57.2 (14)	44.9 (13)	45.0 (14)	66.3 (5)
Time worked as a nurse, mean (SD), y	27.5 (15)	16.6 (12)	15.6 (13)	36.7 (9)
Experience				
Are very or moderately satisfied with nursing as a career choice	3197 (79)	239 (71)	926 (65)	2032 (90)
Retired earlier than planned	NA	NA	NA	855 (37)
Searched for work in the last year				
In health care	1035 (26)	83 (25)	719 (51)	233 (10)
Not in health care	397 (10)	126 (37)	143 (10)	128 (6)
Have not searched for work	2545 (64)	127 (38)	540 (39)	1878 (84)
Likelihood of returning to work as a nurse				
Very likely	687 (17)	66 (20)	534 (38)	87 (4)
Somewhat likely	738 (19)	53 (16)	405 (29)	280 (13)
Somewhat unlikely	805 (20)	78 (23)	244 (18)	483 (21)
Very unlikely	1741 (44)	136 (41)	215 (15)	1390 (62)

Abbreviation: NA, not applicable.

^a^ Missing responses were minimal (<5%). Age was missing in 201 respondents (5%). In conducting a missingness analysis, respondents with missing age had slightly fewer years of experience, were not currently employed, and were likely to return to work.

^b^ Unless otherwise indicated.

Most RNs not currently employed recently searched for work in health care (719 [51%]) and were likely to return to work (939 [67%]). RNs employed outside health care were less likely to return to RN work, although 66 (20%) said they are very likely to return. Among retired RNs, 855 (37%) reported retiring earlier than planned. Most retired RNs were unlikely to return to work and had not recently searched for work; thus, they were excluded from the remainder of analyses.

Most nonretired RNs said adequate staffing (1113 [65%]), flexible scheduling (1006 [59%]), and better wages and benefits (1013 [59%]) would increase their likelihood of returning (Table 2). RNs of all ages identified these top factors as important to them, but they were particularly salient to younger RNs. Only 141 RNs (8%) reported that nothing would bring them back.

**Table 2.** Organizational Factors That Would Increase Nonretired Nurses’ Likelihood of Returning to Work as a Nurse Overall and by Age Category^a^

Organizational factor	No. (%) of nonretired nurses					
	Total (n = 1778)	By age categories, y				
		≤30 (n = 238)	31-40 (n = 509)	41-50 (n = 274)	51-60 (n = 287)	≥61 (n = 282)
Adequate staffing or a manageable workload	1113 (65)	175 (74)	356 (70)	193 (71)	167 (58)	137 (49)
Flexible scheduling	1006 (59)	154 (65)	328 (64)	168 (62)	143 (50)	125 (45)
Better wages and benefits	1013 (59)	168 (71)	355 (70)	164 (60)	143 (50)	92 (33)
Better opportunities for career advancement	536 (31)	85 (36)	241 (47)	88 (32)	47 (16)	26 (9)
Other	382 (22)	34 (14)	93 (18)	56 (21)	88 (31)	86 (31)
I want to work in nursing but cannot find employment	138 (8)	22 (9)	30 (6)	22 (8)	27 (9)	26 (9)
Nothing would bring me back to work in nursing	141 (8)	19 (8)	29 (6)	17 (6)	32 (11)	36 (13)

^a^ This analysis is based on a sample size of 1778 nurses who are either not currently employed or employed in work outside the health care sector. Column percentages do not sum to 100% because nurse respondents were instructed to select all that apply. Missingness for organizational factors data was minimal (<5% missing for variables). Among the 1778 nonretired nurses, 142 (8%) had missing data on age and thus are not reported in the age categories. In conducting a missingness analysis, respondents with missing age had slightly fewer years of experience, were not currently employed, and were likely to return to work.

## Discussion

In this cross-sectional study of 4043 RNs who recently left a hospital staff nurse job, we found opportunities for reattracting an existing RN workforce if hospitals are willing to address organizational issues driving RNs away. Many nonretired RNs, particularly those not currently employed, reported being very likely to return to work. The top factors to increase their likelihood of returning were adequate staffing, flexible scheduling, and better wages or benefits.

Inadequate staffing is a top reason RNs leave,^4^ and as this study suggests, improving staffing adequacy could bring them back. Opening more RN positions at the bedside and providing flexible scheduling arrangements are promising strategies to reattract RNs.^5^ A limitation of this study is that we were only able to survey RNs who maintained a license; thus, the sample may be biased toward RNs who are more likely to return to work.

Most RNs are satisfied with nursing as a career but dissatisfied with their employers. High RN turnover is a solvable crisis that can be remedied by employers because the reasons RNs leave are the same reasons they would return, if addressed.^2,4^

## Notes

Supplement 1. **eAppendix. **Study Methods

Supplement 2.
Data Sharing Statement
