# Supplementary material for: Organizational Factors to Reattract Nurses to Hospital Employment
Source: JAMA Netw Open. 2026 Feb 9;9(2):e2556570. doi: 10.1001/jamanetworkopen.2025.56570 (PMC12887740; doi:10.1001/jamanetworkopen.2025.56570)
Supplement: Supplement 1. — eAppendix. Study Methods [file jamanetwopen-e2556570-s001.pdf]

Supplemental Online Content

Lasater KB, McHugh MD, Muir KJ. Organization factors to reattract nurses to hospital employment. *JAMA Netw Open*. 2026;9(2):e2556570.
doi:10.1001/jamanetworkopen.2025.56570

eAppendix. Study Methods

This supplemental material has been provided by the authors to give readers additional information about their work.

© 2026 Lasater KB et al. *JAMA Network Open.*

eAppendix. Study Methods

Nurses4All Survey Data

Nurses from 10 states (CA, FL, IL, LA, NJ, NM, NY, OR, PA, WA) were identified via state licensure lists of registered nurses and invited to participate in the Nurses4All survey. The survey was conducted via email from December 2023 to March 2024 in collaboration with the National Council of State Boards of Nursing which is an organization with access to registered nurse licensure lists. Nurse respondents to the survey indicated consent through respondents' participation in the study, which was voluntary.

Since our survey approach[1] identifies potential respondents based on licensure data, as opposed to employment data, we are uniquely able to identify a large sample of registered nurses who have maintained their license to practice despite not being employed in a healthcare job. The survey design builds on over 20 years of prior multi-state surveys of nurses with the methodology detailed elsewhere.[1] It is impossible to compute a survey response rate for on our sample of interest (nurses not working in healthcare jobs) since there is no available information to compute the denominator of the sampling frame.

Study Sample

For this study, the nurse respondents were included in the analytic sample if they met the following criteria: (1) they reported their employment status was either 'employed, but not in healthcare,' 'not currently employed,' or 'retired,' (2) the most recent healthcare job they worked was as a direct care staff nurse in a hospital, (2) they left that hospital job within the last 5 years (2019-2023). Based on these criteria, we had an analytic sample of 4,043 registered nurses.

Study Variables

Nurses responded to questions about their employment and career experiences, including how satisfied they were with nursing as a choice of career (response options: 4-point Likert scale ranging from "very satisfied" to "very disappointed"), whether they searched for work in the last year (response options: "yes, in healthcare," "yes, not in healthcare" or "no"), and among the retired nurses, we asked whether they retired earlier than they would have liked (response options: "yes" and "no").

All nurses were asked, "How likely are you to return to work as a nurse?" Responses of "very likely" and "somewhat likely" were categorized as "likely to return"; while responses of "somewhat unlikely" and "very unlikely" were categorized as "unlikely to return."

Nurses responded to the questions "what would increase your likelihood of return to work as a nurse" by selecting all that apply for a list of options. Demographic factors included age and years worked as a nurse.

eReference

1. Lasater, K. B., Jarrín, O. F., Aiken, L. H., McHugh, M. D., Sloane, D. M., & Smith, H. L. (2019). A methodology for studying organizational performance: a multistate survey of front-line providers. Medical Care, 57(9), 742-749.

© 2026 Lasater KB et al. *JAMA Network Open.*
