# Supplementary material for: Organizational Factors to Reattract Nurses to Hospital Employment
Source: JAMA Netw Open. 2026 Feb 9;9(2):e2556570. doi: 10.1001/jamanetworkopen.2025.56570 (PMC12887740; doi:10.1001/jamanetworkopen.2025.56570)
Supplement: Supplement 2. — Data Sharing Statement [file jamanetwopen-e2556570-s002.pdf]

Data Sharing Statement

Lasater. Organizational Factors to Reattract Nurses to Hospital Employment. *JAMA Netw Open*. Published February 09, 2026. doi:10.1001/jamanetworkopen.2025.56570

Data
Data available: No
